# Cardiovascular Benefits of Glucagon-Like Peptide-1 (GLP-1) Receptor Agonists in Type 2 Diabetes Mellitus With Atherosclerotic Cardiovascular Disease: A Systematic Review of Randomized Controlled Trials

**DOI:** 10.7759/cureus.89514

**Published:** 2025-08-06

**Authors:** Maria Ahmad, Ayesha Sikandar, Abdul Aziz, Wisam Bachar Al Sumodi, Aakash Hans, Muhammad Usman

**Affiliations:** 1 Internal Medicine, The Royal Wolverhampton National Health Service (NHS) Trust, Wolverhampton, GBR; 2 Internal Medicine, Karachi Medical and Dental College, Karachi, PAK; 3 Internal Medicine, Latin American School of Medicine, Havana, CUB; 4 Internal Medicine, Ivane Javakhishvili Tbilisi State University, Tbilisi, GEO; 5 Research, Henry Ford Health System, Detroit, USA; 6 Internal Medicine, White River Health System, Batesville, USA; 7 Internal Medicine, Jinnah Hospital, Lahore, PAK

**Keywords:** ascvd, cardiovascular outcomes, glp-1 receptor agonists, heart failure, semaglutide, systematic review, type 2 diabetes

## Abstract

This systematic review evaluates the cardiovascular effects of glucagon-like peptide-1 receptor agonists (GLP-1 RAs) in adults with type 2 diabetes mellitus (T2DM) and established atherosclerotic cardiovascular disease, chronic kidney disease, or heart failure (HF). A comprehensive literature search across four major databases identified eight eligible studies, including randomized controlled trials and prespecified or pooled post-hoc analyses. The findings demonstrate consistent cardiovascular benefits of GLP-1 RAs, particularly semaglutide and exenatide, with notable reductions in major adverse cardiovascular events, cardiovascular mortality, and HF-related outcomes. Mechanistically, these benefits may be attributed to anti-inflammatory effects, improved endothelial function, and metabolic improvements such as weight loss and blood pressure reduction. Despite some heterogeneity across subgroups and study designs, the overall evidence supports the integration of GLP-1 RAs into cardiovascular risk management for patients with T2DM.

## Introduction and background

Type 2 diabetes mellitus (T2DM) is a chronic metabolic disorder characterized by insulin resistance and progressive beta-cell dysfunction, affecting an estimated 537 million adults globally as of 2021, a number projected to rise to 783 million by 2045 [1]. Beyond glycemic abnormalities, T2DM is a well-established and potent risk factor for atherosclerotic cardiovascular disease (ASCVD), which remains the leading cause of morbidity and mortality among individuals with diabetes. Epidemiological data suggest that adults with T2DM have approximately two to four times higher risk of experiencing major adverse cardiovascular events (MACE), such as myocardial infarction, stroke, HF, and cardiovascular (CV) death, compared to those without diabetes [2]. As such, CV risk reduction has become a critical therapeutic goal in the comprehensive management of T2DM.

In recent years, glucagon-like peptide-1 receptor agonists (GLP-1 RAs) have emerged as a novel class of antidiabetic agents that extend benefits beyond glycemic control. These agents mimic the action of endogenous GLP-1, enhancing glucose-dependent insulin secretion, inhibiting glucagon release, and promoting satiety and weight loss [3]. Notably, large-scale CV outcome trials have demonstrated the cardioprotective potential of GLP-1 RAs in high-risk diabetic populations. Building upon early trials like LEADER, SUSTAIN-6, and REWIND, several recent high-powered trials, such as SOUL [4] (N=9,650), FLOW [5] (N=3,533), and the STEP-HFpEF program [6] (N=1,145), have refined our understanding of semaglutide’s impact on both CV and cardiorenal outcomes. These newer studies, often referred to as “second-generation trials,” are characterized by enhanced design rigor, broader inclusion criteria (e.g., chronic kidney disease (CKD) and HF with preserved ejection fraction (HFpEF)), and expanded endpoints, including NT-proBNP levels, epicardial adipose tissue modulation, and HF-specific metrics. Additionally, some trials have investigated potential synergistic or independent effects when GLP-1 RAs are used alongside agents like sodium-glucose cotransporter-2 (SGLT2) inhibitors.

Despite the growing body of literature, the evidence surrounding the CV efficacy of GLP-1 RAs, particularly semaglutide and exenatide, in patients with T2DM and established ASCVD remains scattered across multiple trials with varying outcome measures and study designs. To address this gap, we conducted a systematic review of high-quality randomized controlled trials (RCTs) to synthesize the current evidence and provide clinicians with consolidated insights for CV risk stratification and treatment selection in this high-risk population.

## Review

Materials and methods

Search Strategy and Eligibility Criteria

This systematic review was conducted following the Preferred Reporting Items for Systematic Reviews and Meta-Analyses (PRISMA) 2020 guidelines [7]. A comprehensive literature search was carried out using PubMed (MEDLINE), Embase, Cochrane CENTRAL, and Scopus to identify relevant studies published in the last one year (from June 2024 to June 2025). The search strategy employed a combination of MeSH terms and free-text keywords, including “GLP-1 receptor agonists”, “semaglutide”, “exenatide”, “cardiovascular outcomes”, “major adverse cardiovascular events”, “type 2 diabetes”, and “atherosclerotic cardiovascular disease”. Filters were applied to restrict results to RCTs, human studies, and English-language articles. Duplicate records were removed, and titles and abstracts were screened independently by two reviewers. Full-text articles were assessed for eligibility based on predefined inclusion and exclusion criteria.

Inclusion and Exclusion Criteria

We included RCTs and prespecified or pooled post-hoc analyses of RCTs that investigated the effects of GLP-1 RAs on CV outcomes in adult patients with T2DM and established ASCVD, CKD, or HF. Eligible studies were required to report outcomes such as MACE, CV mortality, hospitalization for HF (HHF), or validated surrogate endpoints like NT-proBNP or NYHA functional class. Studies conducted in non-human models, trials not reporting relevant CV outcomes, and those involving pediatric populations or type 1 diabetes mellitus were excluded.

PICO Framework

The study was designed using the PICO model [8]: population (adults with T2DM and ASCVD, CKD, or HF), intervention (GLP-1 RAs including semaglutide and exenatide), comparator (placebo or standard care), and outcomes (CV-related outcomes such as MACE, CV mortality, HF hospitalization, and surrogate markers, e.g., NT-proBNP and NYHA class).

Data Extraction and Risk of Bias Assessment

From each included study, data were extracted on study design, sample size, patient population, intervention and comparator details, follow-up duration, and primary CV outcomes. Data extraction was conducted independently by two authors and verified by a third reviewer to ensure accuracy. The risk of bias for each RCT was assessed using the Cochrane Risk of Bias 2 (RoB 2, Cochrane Collaboration, London, UK) tool [9]. Criteria such as randomization, allocation concealment, blinding of participants and outcome assessors, completeness of outcome data, and selective reporting were evaluated.

Study Selection and Review Process

Eight studies were finalized for inclusion based on relevance, recency, and methodological quality. These included large multicenter RCTs and pooled post-hoc analyses that together represent a broad spectrum of patients with high cardiometabolic risk. A PRISMA flow diagram outlining the study selection process has been included in the supplementary materials. Discrepancies during the selection or data extraction process were resolved through consensus discussion.

Data Analysis and Synthesis

Due to the heterogeneity in outcome measures and population subgroups, a formal meta-analysis was not conducted. Instead, a qualitative synthesis of the results was performed. Findings from the included trials were summarized narratively, with a particular focus on hazard ratios, odds ratios, or effect size estimates for primary endpoints such as MACE, CV death, and HF-related events. Subgroup effects, such as differences based on baseline NYHA class, ejection fraction, obesity, or SGLT2 inhibitor use, were also examined descriptively. The consistency and direction of the findings across trials were used to determine overall trends and implications.

Results

Study Selection Process

**Figure 1:**
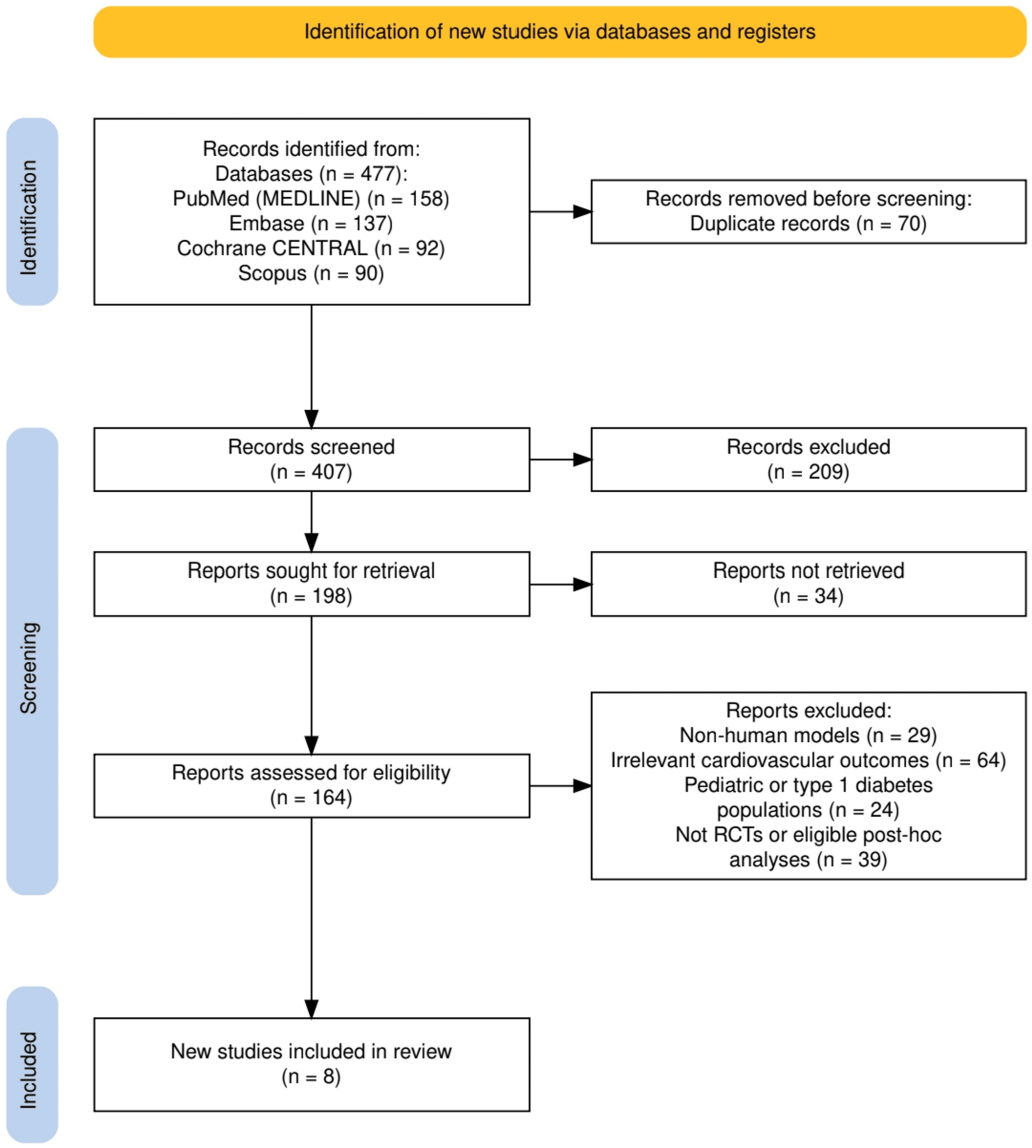
PRISMA flowchart representing the study selection process Prisma: Preferred Reporting Items for Systematic Reviews and Meta-Analyses, RCT: randomized controlled trial

As illustrated in Figure 1, a total of 477 records were identified across four databases, with 70 duplicates removed prior to screening. Following a rigorous screening and eligibility assessment, 156 studies were excluded for reasons such as non-human models (n=29), irrelevant CV outcomes (n=64), pediatric/type 1 diabetes populations (n=24), or ineligible study design (n=39). Ultimately, eight studies met the inclusion criteria and were incorporated into this systematic review.

Characteristics of the Selected Studies

As shown in Table 1, eight eligible studies were included in this systematic review, comprising RCTs and prespecified or pooled post-hoc analyses. These studies investigated the CV effects of GLP-1 RAs in adults with T2DM and comorbid ASCVD, CKD, or HF. The trials consistently reported on key outcomes such as MACE, CV mortality, HF hospitalization, and surrogate markers like NT-proBNP and NYHA class.

**Table 1:** Summary of the characteristics of the included studies in the review RCT: randomized controlled trial, T2DM: type 2 diabetes mellitus, ASCVD: atherosclerotic cardiovascular disease, CKD: chronic kidney disease, SC: subcutaneous, CV: cardiovascular, HFpEF: heart failure with preserved ejection fraction, HF: heart failure, MACE: major adverse cardiovascular events, HR: hazard ratio, CI: confidence interval, p: p-value, SGLT2i: sodium-glucose cotransporter-2 inhibitor, ↓: decrease, ↑: increase, ETR: estimated treatment ratio, NT-proBNP: N-terminal pro-B-type natriuretic peptide, NYHA: New York Heart Association (functional classification), OR : odds ratio, LVEF: left ventricular ejection fraction, HHF: hospitalization for heart failure, p-int: p-value for interaction, EQW: exenatide once weekly

Study (author, year, trial name)	Study design	Population	Intervention	Comparator	Outcome(s)
McGuire et al., 2025 (SOUL Trial) [10]	RCT, double-blind, placebo-controlled	N=9,650 adults ≥50 yrs with T2DM and ASCVD or CKD	Oral semaglutide (up to 14 mg daily)	Placebo	MACE: HR 0.86 (95% CI, 0.77-0.96), p=0.006
Perkovic et al., 2024 (FLOW Trial) [5]	RCT, double-blind, placebo-controlled	N=3,533 with T2DM and CKD	SC semaglutide 1.0 mg weekly	Placebo	CV death: HR 0.71 (95% CI, 0.56-0.89); MACE HR 0.82
Kosiborod et al., 2024 (SELECT/FLOW/STEP pooled) [11]	Pooled post-hoc analysis of 4 RCTs	N=3,743 with HFpEF from 22,282 total	SC semaglutide (1.0–2.4 mg) weekly	Placebo	CV death or HF event: HR 0.69, p=0.0045
Marx et al., 2025 (SOUL SGLT2i Subgroup) [4]	Subgroup analysis of RCT	N=9,650 with T2DM and ASCVD/CKD ± SGLT2i	Oral semaglutide 14 mg daily	Placebo	MACE HR 0.86 across SGLT2i subgroups
Pratley et al., 2024 (FLOW HF Outcomes) [12]	Prespecified RCT analysis	N=3,533 with T2DM + CKD (19% HF at baseline)	Semaglutide 1.0 mg SC weekly	Placebo	↓HF events/CV death: HR 0.73, p=0.0005
Petrie et al., 2024 (STEP-HFpEF NT-proBNP) [13]	Pooled prespecified analysis of RCTs	N=1,145 with obesity-related HFpEF	Semaglutide 2.4 mg SC weekly	Placebo	↓NT-proBNP: ETR 0.82, p=0.0002
Schou et al., 2024 (STEP-HFpEF NYHA) [6]	Pooled RCT analysis	N=1,145 with obesity-related HFpEF	Semaglutide 2.4 mg SC weekly	Placebo	↑NYHA class: OR 2.20; ↓deterioration: OR 0.36
Neves et al., 2025 (EXSCEL Trial) [14]	Post-hoc analysis of RCT	N=14,752 with T2DM; 4,749 with baseline LVEF	Exenatide (EQW)	Placebo	HHF: HR 0.74 (LVEF ≥40%), HR 1.52 (LVEF <40%); p-int = 0.012. No impact on MACE or CV death

Risk of Bias Assessment

As demonstrated in Table 2, the risk of bias assessment revealed that five of the eight included studies, primarily RCTs, were rated as having a low overall risk of bias using the RoB 2.0 tool. We selected RoB 2.0 because it is a standardized and widely accepted framework specifically designed for evaluating RCTs, focusing on critical domains such as randomization, allocation concealment, blinding, missing outcome data, and selective reporting. This tool allows for a structured and transparent assessment of potential bias that could affect the internal validity of trial results.

**Table 2:** The risk of bias assessment of each of the included studies.

Study (Author, Year, Trial Name)	Tool Used	Randomization Process	Deviations from Intended Interventions	Missing Outcome Data	Measurement of Outcome	Selection of Reported Results	Overall Risk of Bias
McGuire DK et al., 2025 (SOUL Trial) [10]	RoB 2.0	Low Risk	Low Risk	Low Risk	Low Risk	Low Risk	Low
Perkovic V et al., 2024 (FLOW Trial) [5]	RoB 2.0	Low Risk	Low Risk	Low Risk	Low Risk	Low Risk	Low
Kosiborod MN et al., 2024 (SELECT/FLOW/STEP pooled) [11]	ROBINS-I	Moderate Risk	Moderate Risk	Low Risk	Low Risk	Moderate Risk	Moderate
Marx N et al., 2025 (SOUL SGLT2i Subgroup) [4]	ROBINS-I	Moderate Risk	Moderate Risk	Low Risk	Low Risk	Moderate Risk	Moderate
Pratley RE et al., 2024 (FLOW HF Outcomes) [12]	RoB 2.0	Low Risk	Low Risk	Low Risk	Low Risk	Low Risk	Low
Petrie MC et al., 2024 (STEP-HFpEF NT-proBNP) [13]	RoB 2.0	Low Risk	Low Risk	Low Risk	Low Risk	Low Risk	Low
Schou M et al., 2024 (STEP-HFpEF NYHA) [6]	RoB 2.0	Low Risk	Low Risk	Low Risk	Low Risk	Low Risk	Low
Neves JS et al., 2025 (EXSCEL Trial) [14]	ROBINS-I	Moderate Risk	Moderate Risk	Moderate Risk	Low Risk	Moderate Risk	Moderate

In contrast, three studies, comprising pooled or post-hoc subgroup analyses, were assessed using the Risk of Bias In Non-randomized Studies of Interventions tool (ROBINS-I), which is better suited for evaluating studies without randomized designs. These studies showed a moderate risk of bias, particularly in domains related to deviations from intended interventions and selective reporting. This methodological distinction highlights the greater robustness and reliability of the primary RCTs included in this review, as well as the need for cautious interpretation of findings from post-hoc analyses.

Discussion

Our systematic review synthesizing data from eight high-quality RCTs and pooled analyses demonstrates that GLP-1 RAs, particularly semaglutide, offer meaningful CV benefits in patients with T2DM, especially those with coexisting ASCVD, CKD, or HFpEF. In the SOUL trial by McGuire et al. [10], oral semaglutide significantly reduced MACE (HR 0.86, p=0.006), an effect mirrored in the FLOW trial by Perkovic et al. [5], where semaglutide lowered both CV death and MACE risk. Moreover, semaglutide improved surrogate outcomes such as NT-proBNP levels and NYHA class in patients with obesity-related HFpEF, as shown by Petrie et al. [13] and Schou et al. [6]. Notably, the EXSCEL trial [14] highlighted a differential effect of exenatide based on left ventricular ejection fraction, suggesting a potential class effect modulated by cardiac function. Collectively, these findings underscore the evolving role of GLP-1 RAs as both glucose-lowering and cardioprotective agents in high-risk diabetic populations.

When compared with earlier landmark trials such as LEADER (liraglutide) [15], SUSTAIN-6 (semaglutide) [16], and REWIND (dulaglutide) [17], our findings affirm the cardioprotective potential of GLP-1 RAs. However, this review builds upon those foundational results by focusing specifically on newer formulations, including oral semaglutide, and expanded patient populations such as those with CKD, HFpEF, and those receiving concomitant SGLT2 inhibitors [18]. Unlike some earlier trials, which primarily assessed atherosclerotic outcomes, newer studies like FLOW and STEP-HFpEF evaluated HF-specific outcomes and surrogate markers, enhancing our understanding of therapeutic versatility. Importantly, the EXSCEL trial’s [14] post-hoc findings diverge from the consistent benefits seen with semaglutide, highlighting a possible variation in class effects, particularly in patients with reduced ejection fraction. These nuanced comparisons emphasize both the progress made and the ongoing need for tailored CV risk reduction strategies in T2DM management.

GLP-1 RAs exert CV benefits through several biologically plausible mechanisms that extend beyond glycemic control. These agents have demonstrated anti-inflammatory effects, reducing levels of pro-inflammatory cytokines and markers such as CRP, which are implicated in the pathogenesis of atherosclerosis [19]. Additionally, GLP-1 RAs improve endothelial function by enhancing nitric oxide bioavailability and reducing oxidative stress, thereby promoting vascular relaxation and reducing arterial stiffness. Their ability to induce significant weight loss and lower blood pressure further contributes to CV risk reduction, especially in patients with obesity or metabolic syndrome [20]. GLP-1 RAs also possess natriuretic properties, promoting sodium excretion and thus relieving volume overload, a critical factor in HF management. Moreover, preclinical studies suggest direct cardioprotective effects via activation of GLP-1 receptors in myocardial and vascular tissues, leading to reduced myocardial apoptosis, fibrosis, and ischemia-reperfusion injury. Together, these mechanistic pathways support the observed clinical outcomes in CV event reduction [21].

Subgroup analyses across the included studies reveal important heterogeneity in CV outcomes, highlighting the need for personalized therapeutic approaches. In the EXSCEL trial [14], exenatide reduced HHF only in patients with preserved ejection fraction (LVEF ≥40%) but not in those with reduced ejection fraction (LVEF <40%), indicating a possible divergence in efficacy between HF phenotypes. Similarly, outcomes in the STEP-HFpEF program [6] were more favorable in patients with lower baseline NYHA class, suggesting that earlier-stage HF may be more modifiable with GLP-1 RAs. The SOUL subgroup analysis [10] showed consistent CV benefits of oral semaglutide across SGLT2i use, supporting potential synergy when used in combination therapy. Furthermore, obesity and CKD presence appear to enhance responsiveness, as seen in the FLOW [12] and STEP trials [13], where semaglutide provided substantial benefit in reducing CV death, HF events, and NT-proBNP levels in patients with these comorbidities. These findings underscore the importance of tailoring therapy based on patient phenotype and comorbidity profile.

This review has several notable strengths that enhance its reliability and clinical relevance. It exclusively includes RCTs and prespecified subgroup analyses, which offer the highest level of evidence and superior methodological rigor compared to observational studies. Unlike observational designs, which are more susceptible to confounding, selection bias, and unmeasured variables, RCTs provide a controlled environment where causal inferences are more robust. The data extracted are recent and encompass a wide spectrum of clinically relevant outcomes, including MACE, CV death, HHF, and functional biomarkers such as NT-proBNP levels and NYHA class. The consistency of CV benefits observed across diverse trials further strengthens the credibility of these findings.

However, the review is not without limitations. While the majority of included studies were RCTs, several were post-hoc or pooled subgroup analyses, which carry an inherent risk of bias. These analyses are often exploratory and may lack prespecified hypotheses or statistical corrections for multiple comparisons, thereby increasing the likelihood of chance findings. Additionally, subgroup results can be influenced by residual confounding, imbalanced covariates, or selective outcome reporting, factors that can compromise internal validity and skew the interpretation of treatment effects. The duration of follow-up was also relatively short in several trials, potentially underestimating long-term outcomes and sustainability of benefit. Furthermore, the generalizability of findings may be limited due to the underrepresentation of minority populations and variations in the definitions and adjudication of composite endpoints like MACE or HF-related outcomes. These limitations must be acknowledged when applying these results to broader, more diverse patient populations.

The clinical implications of our findings are profound, especially for managing high-risk patients with T2DM and established ASCVD, CKD, or HFpEF. These data support the prioritization of GLP-1 RAs, particularly semaglutide, as a frontline therapy for CV risk reduction in this population [22]. Clinicians should consider initiating GLP-1 RAs early in the disease course, ideally in conjunction with comprehensive cardiometabolic screening, to identify individuals who stand to benefit most. The favorable outcomes seen in patients with concomitant SGLT2 inhibitor use further suggest a synergistic effect, advocating for combined use in appropriate patients to target multiple pathophysiological pathways [23]. These insights should prompt updates in clinical guidelines and stimulate broader implementation of GLP-1 RAs in routine practice to reduce the burden of CVD in diabetes.

## Conclusions

Our systematic review demonstrates that GLP-1 RAs, particularly semaglutide and exenatide, provide significant CV benefits in patients with T2DM and established ASCVD, CKD, or HFpEF. These agents consistently reduce MACE, improve HF outcomes, and enhance functional status, especially in high-risk populations. By synthesizing robust, recent RCT data and meaningful subgroup insights, our findings support the integration of GLP-1 RAs as essential components of cardiometabolic care, not only for glycemic control, but also for reducing CV morbidity and mortality. Looking ahead, future research should focus on long-term follow-up to evaluate the durability of these benefits, explore comparative effectiveness among GLP-1 RAs, and assess their impact in more diverse and underrepresented populations to further guide clinical decision-making and refine treatment guidelines.
